# Author Correction: Genetic analysis of seed traits in *Sorghum bicolor* that affect the human gut microbiome

**DOI:** 10.1038/s41467-022-34544-7

**Published:** 2022-11-08

**Authors:** Qinnan Yang, Mallory Van Haute, Nate Korth, Scott E. Sattler, John Toy, Devin J. Rose, James C. Schnable, Andrew K. Benson

**Affiliations:** 1 Department of Food Science and Technology, University of Nebraska, Lincoln, NE USA; 2 Nebraska Food for Health Center, University of Nebraska, Lincoln, NE USA; 3 Complex Biosystems Graduate Program, University of Nebraska, Lincoln, NE USA; 4 Wheat, Sorghum and Forage Research Unit, USDA-ARS, Lincoln, NE USA; 5 Department of Agronomy and Horticulture, University of Nebraska, Lincoln, NE USA; 6 Center for Plant Science Innovation, University of Nebraska, Lincoln, NE USA

**Keywords:** Quantitative trait, Microbiome, Agricultural genetics, Plant genetics

Correction to: *Nature Communications* 10.1038/s41467-022-33419-1, published online 26 September 2022

In the original version of this article, the orientation of genetic maps placed on the sorghum chromosomes in Figs. 3a and 4a were inverted relative to their orientation in the published sorghum genome pseudomolecules. The orientation of the genetic maps and the associated labels in Fig. 3b have been corrected in both the PDF and HTML versions of the article. These corrections don’t affect the text and the results reported in Table 1 and Supplementary Table 1.

