# Supplementary material for: Genetic analysis of seed traits in Sorghum bicolor that affect the human gut microbiome
Source: Nat Commun. 2022 Sep 26;13:5641. doi: 10.1038/s41467-022-33419-1 (PMC9513080; doi:10.1038/s41467-022-33419-1)
Supplement: Supplementary file 1 — Supplementary Information [file 41467_2022_33419_MOESM1_ESM.pdf]

Genetic analysis of seed traits in *Sorghum bicolor* that affect the human gut microbiome

Yang *et al.*

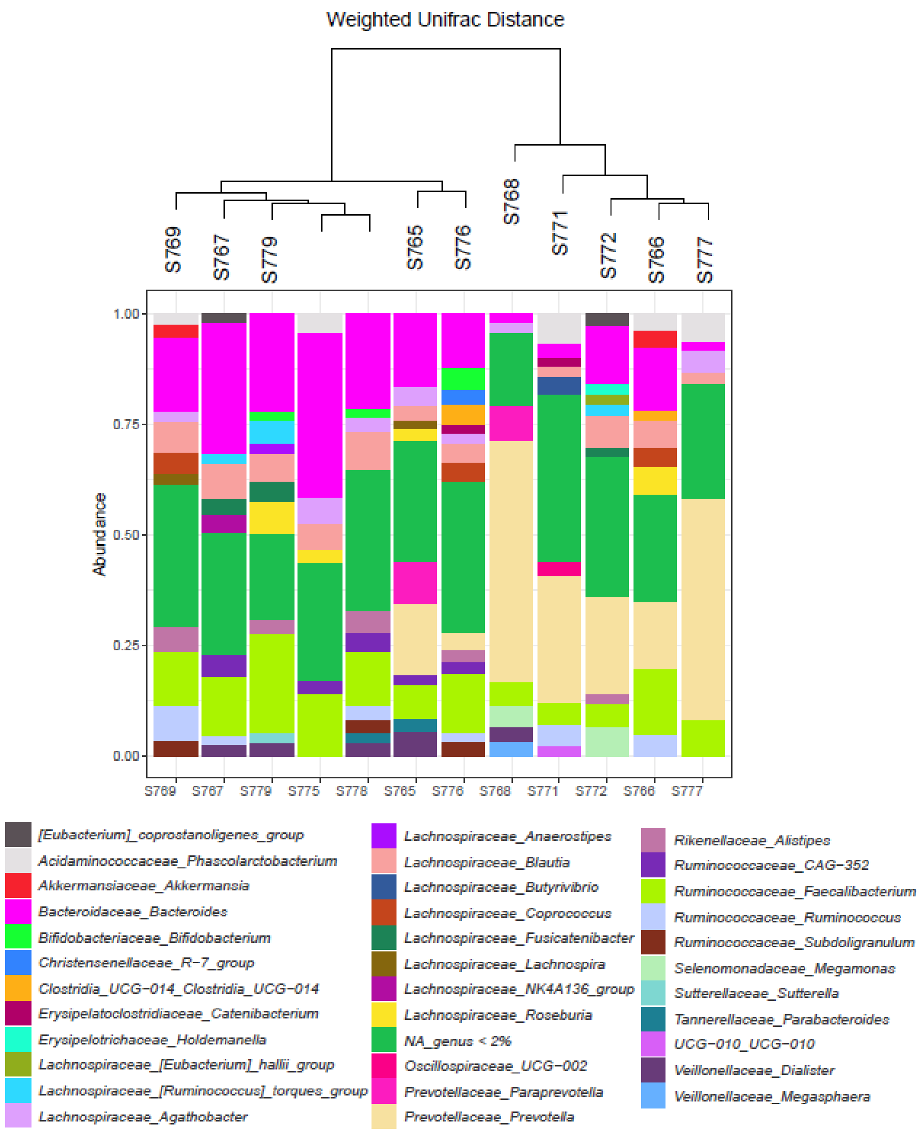

Supplementary Fig. 1. Baseline fecal microbiome composition of 12 different human subjects. Dendrogram was generated using the Ward clustering algorithm with the weighted UniFrac distance matrix of baseline microbiome composition. Stacked bar chart showing the baseline microbiome composition at genus level.

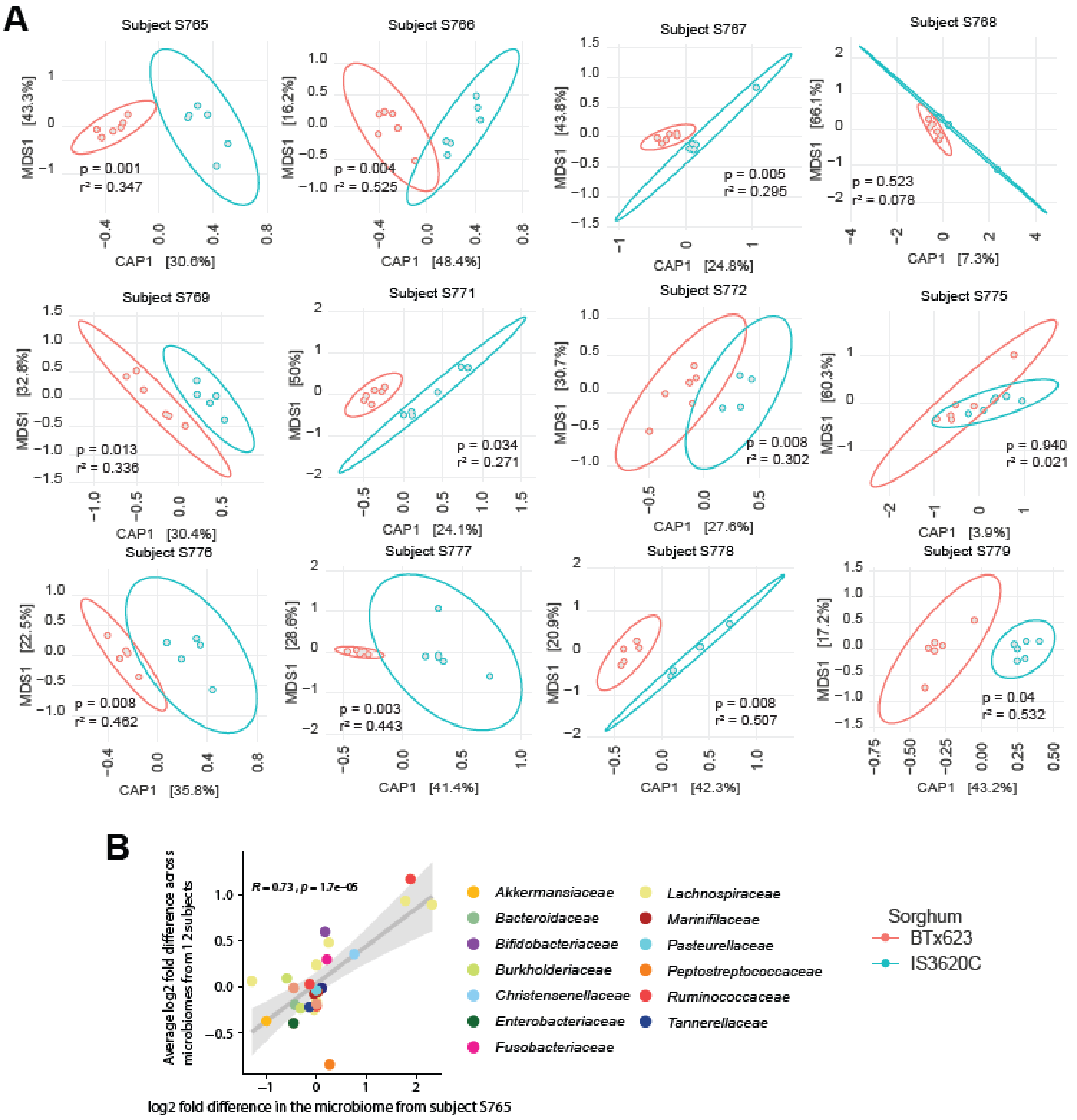

Supplementary Fig. 2. Responsiveness of the S765 microbiome to parental lines. (A) Canonical analysis of principal coordinates (CAP) plot based on Bray-Curtis distance between two parental sorghums in 12 subjects. (*p*-values were calculated using two-sided PERMANOVA). (B) Correlation of the log2 fold difference in the microbiome from subject S765 versus average log2 fold difference for each taxon across microbiomes from 12 subjects. Pearson correlation coefficient and two-tailed *p* values are shown on the figure. Shaded regions represent 95% confidence intervals.

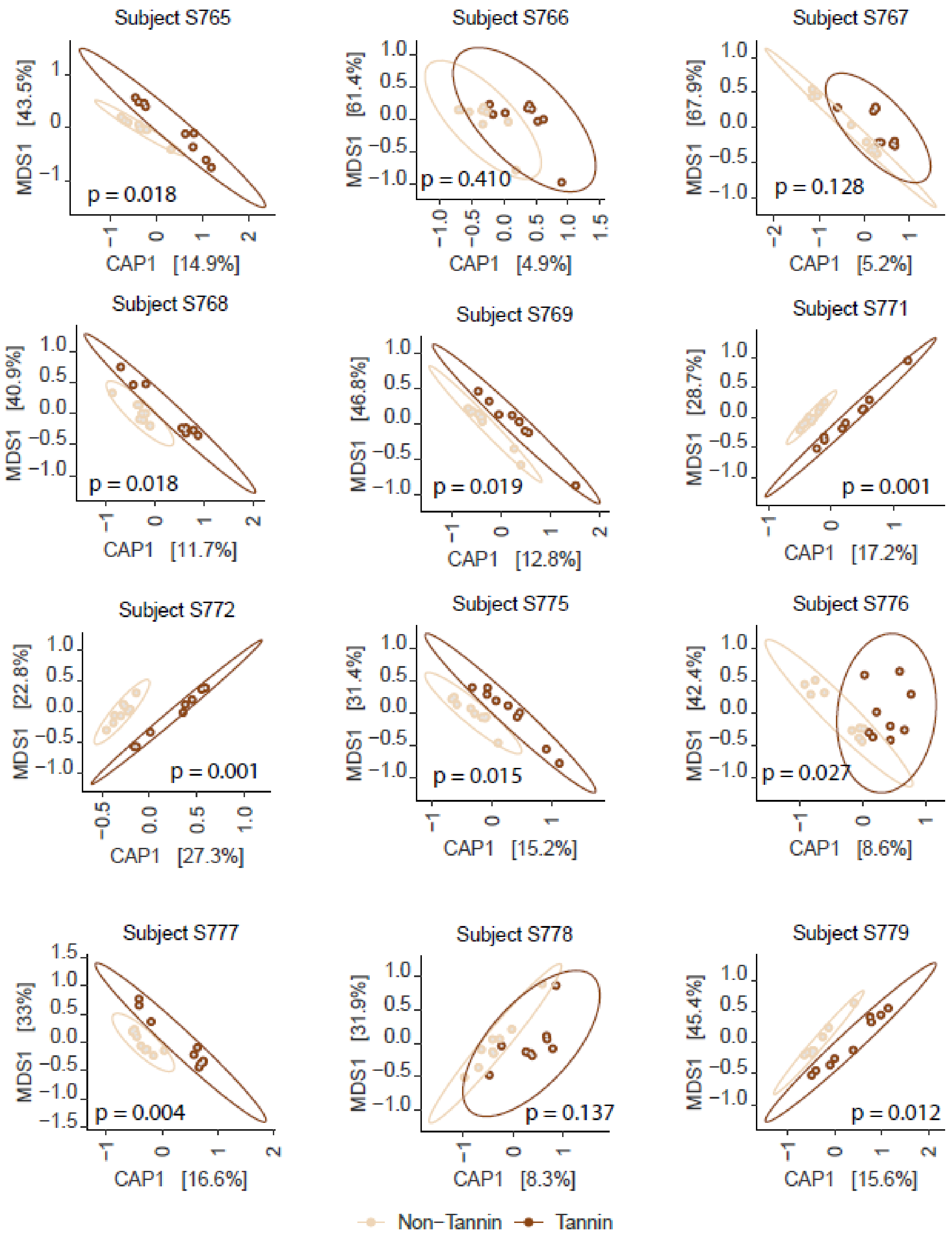

Supplementary Fig. 3. Canonical analysis of principal coordinates (CAP) plot based on Bray-Curtis distance between near iso-genic tannin and non-tannin sorghum in 12 subjects. *P*-values were determined using two-sided PERMANOVA.

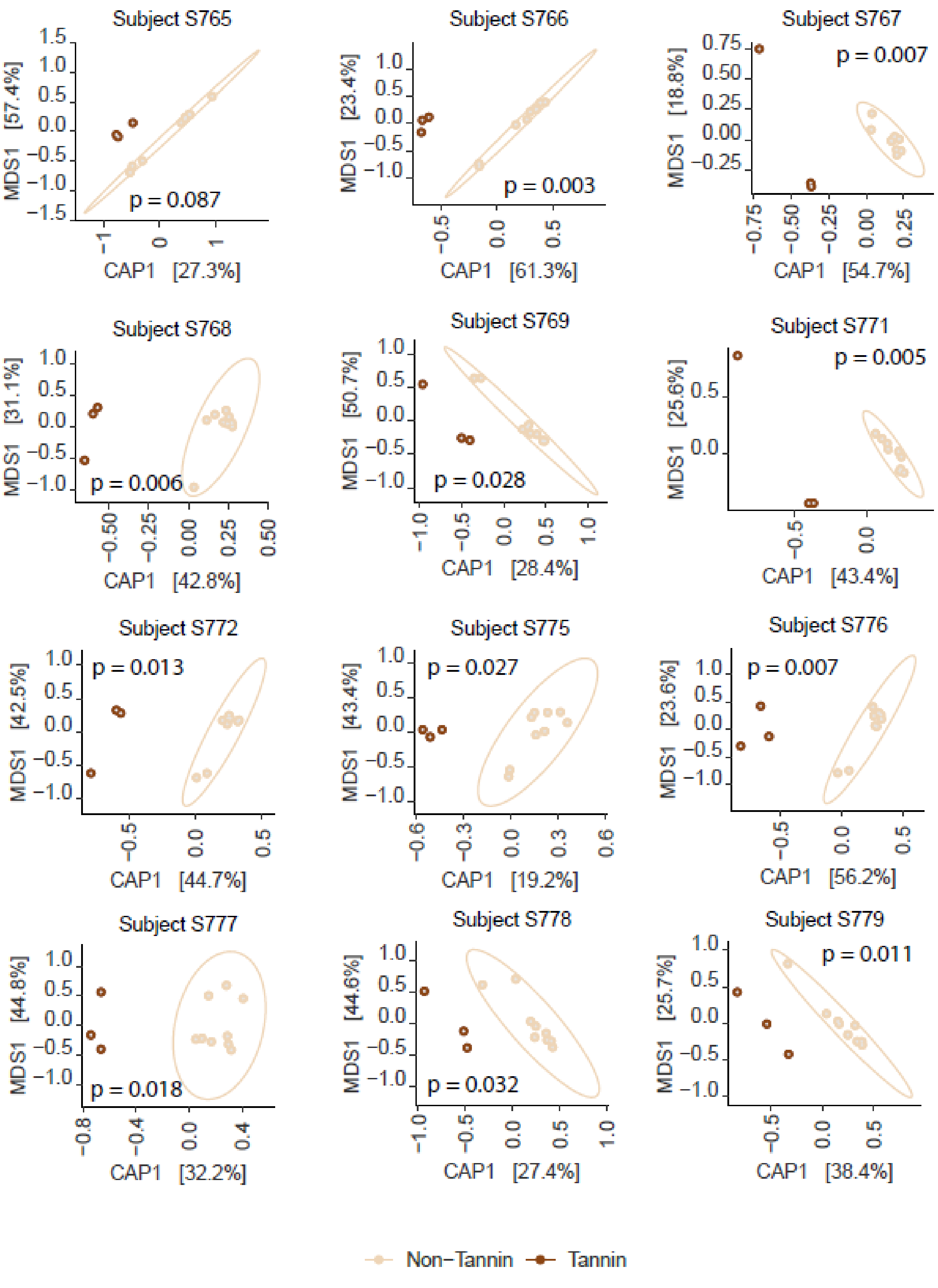

Supplementary Fig. 4. Canonical analysis of principal coordinates (CAP) plot based on Bray-Curtis distance between pools of tannin-positive (*Tan1/Tan2* haplotype) and tannin-negative pools of other haplotypes (*tan-1-b/Tan2, Tan 1/tan-2-c, tan-1-b/tan-2-c*) of sorghum RILs in 12 subjects. *P*-values were determined using two-sided PERMANOVA.

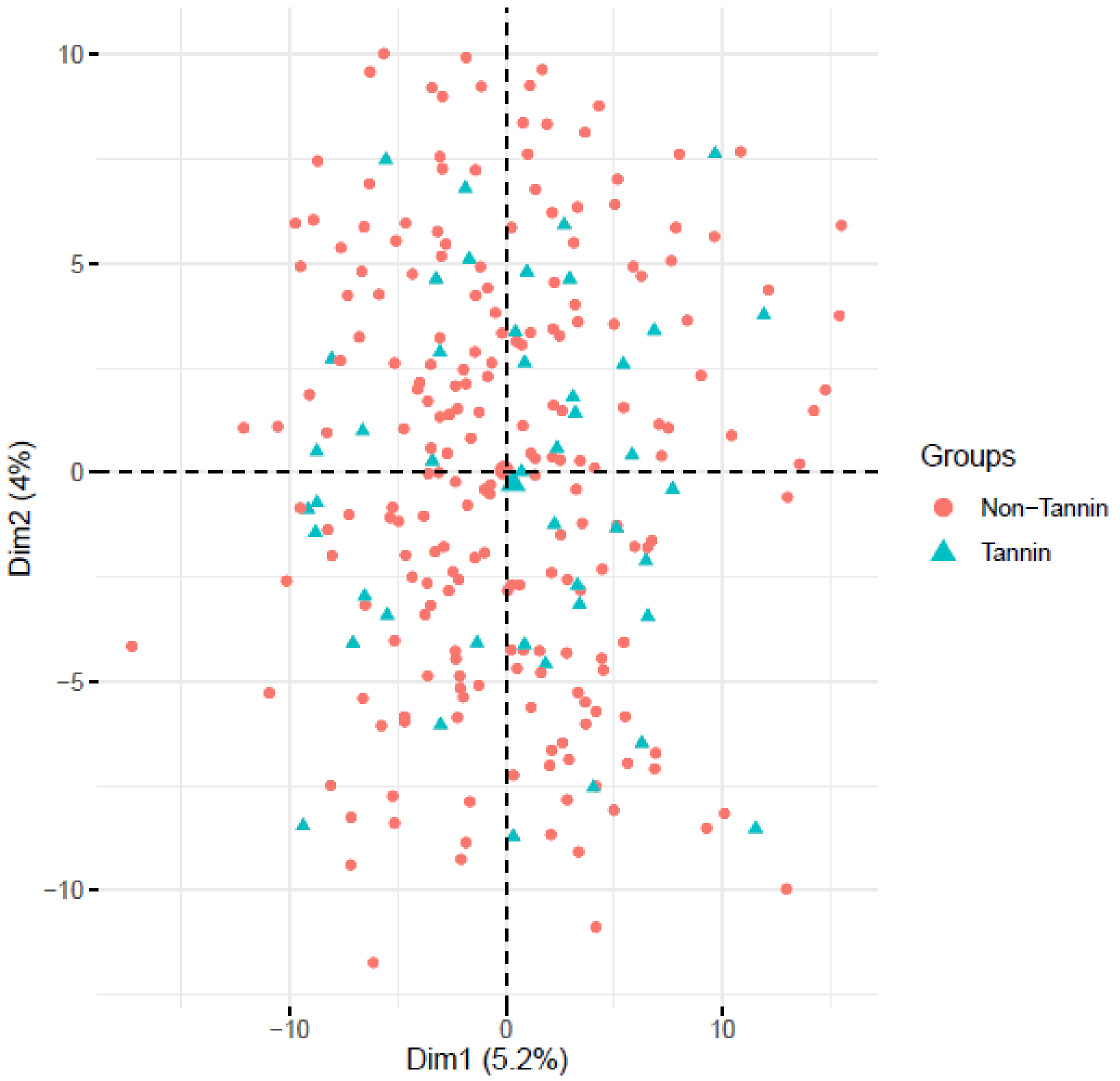

Supplementary Fig. 5. PCA plot of tannin vs non-tannin RILs in the context of total genetic diversity of the RIL population. The plot was developed from the first two PCs based on the SNP markers used for mapping. Each RIL is colored based on the haplotype of alleles at the *Tan1* and *Tan2* loci.

Supplementary Table 1. Significant or suggestive QTLs for microbial taxa and short chain fatty acid traits.

microbial taxa or SCFA	Chromo-some	LOD score	Marker position	*p*	Effect size (%)
Barnesiellaceae_Barnesiella	1	2.77	S1_14765342	0.09	1.54
Ruminococcaceae_Faecalibacterium	2	2.94	S2_7930509	0.057	1.87
Ruminococcaceae_RuminococcaceaeUCG.002	2	3.43	S2_7930509	0.02	1.89
Christensenellaceae_ChristensenellaceaeR.7group	2	4.17	S2_9693506	0.002	1.81
Peptostreptococcaceae_Paeniclostridium	2	3.22	S2_9693506	0.025	1.71
Burkholderiaceae_Sutterella	2	2.87	S2_63835865	0.081	1.21
Prevotellaceae_Paraprevotella	2	4.05	S2_65535874	0.005	1.98
Butyrate	2	4.84	S2_65688971	0.001	2.72
Valerate	2	3.73	S2_65688971	0.005	2.23
Erysipelotrichaceae_Catenibacterium	3	3.01	S3_4044381	0.06	1.04
Lachnospiraceae_Dorea	3	3.61	S3_4044381	0.009	1.53
Lachnospiraceae_Coprococcus3	3	3.88	S3_4044381	0.007	1.89
Christensenellaceae_ChristensenellaceaeR.7group	3	3.63	S3_71508071	0.007	1.16
Lachnospiraceae_Roseburia	3	3.52	S3_71508071	0.015	1.74
Tannerellaceae_Parabacteroides	4	2.74	S4_54983711	0.083	1.62
Peptostreptococcaceae_Paeniclostridium	4	8.64	S4_59462696	0	4.99
Prevotellaceae_Paraprevotella	4	4.01	S4_61161519	0.005	2.25
Christensenellaceae_ChristensenellaceaeR.7group	4	7.37	S4_61304986	0	3.2
Ruminococcaceae_RuminococcaceaeUCG.002	4	4.74	S4_61555802	0	2.23
Ruminococcaceae_Faecalibacterium	4	7.02	S4_61878324	0	3.28
Erysipelotrichaceae_Catenibacterium	4	5.26	S4_61878324	0	2.84
Lachnospiraceae_Roseburia	4	5.41	S4_61878324	0.001	2.77
Lachnospiraceae_Coprococcus1	4	4.09	S4_61878324	0.003	2.38
Valeric	5	3.51	S5_13001287	0.01	1.98
Lachnospiraceae_Roseburia	5	2.86	S5_47691684	0.068	1.62
Lachnospiraceae_Dorea	5	3.19	S5_47691684	0.023	1.48
Lachnospiraceae_Coprococcus3	5	3.04	S5_47691684	0.048	1.28
Lachnospiraceae_Blautia	5	3.07	S5_47691684	0.033	0.81
Butyrate	5	3.08	S5_47691684	0.035	1.75
Propionate	5	3.35	S5_54776076	0.022	1.78
Lachnospiraceae_Roseburia	6	3.38	S6_51140222	0.023	1.88
Lachnospiraceae_Dorea	6	2.67	S6_53024770	0.084	0.93
Clostridiaceae1_NA	6	3.42	S6_58531384	0.017	0.02
Desulfovibrionaceae_Mailhella	7	3.2	S7_1861602	0.035	1.4
Lachnospiraceae_Roseburia	9	2.96	S9_5394048	0.053	1.65
Ruminococcaceae_Butyricicoccus	10	4.14	S10_3639770	0.003	1.16
Lachnospiraceae_Coprococcus3	10	3.5	S10_4146463	0.024	1.79

P value was calculated through 1000 permutations.
