# Supplementary material for: Genetic analysis of seed traits in Sorghum bicolor that affect the human gut microbiome
Source: Nat Commun. 2022 Sep 26;13:5641. doi: 10.1038/s41467-022-33419-1 (PMC9513080; doi:10.1038/s41467-022-33419-1)
Supplement: Supplementary file 3 — Description of Additional Supplementary Files [file 41467_2022_33419_MOESM3_ESM.pdf]

Supplementary Data 1. Shapiro–Wilk test results on 84 taxa.

Supplementary Data 2. Genus table across all RILs.

Supplementary Data 3. Seed color phenotype across all RILs.

Supplementary Data 4. Tannin content across all RILs.

Supplementary Data 5. Seed color, tannin content, and relative abundance of selected bacterial genus across all RILs by haplotype of markers linked to *Tan1* and *Tan2*.

Supplementary Data 6. Random selected RILs that tested across microbiomes from 12 subjects based on haplotype of markers linked to *Tan1* and *Tan2*.

Supplementary Data 7. Impactful sequence polymorphism of candidate genes between BTx623 and IS3620C.
