# Supplementary material for: Genetic analysis of seed traits in Sorghum bicolor that affect the human gut microbiome
Source: Nat Commun. 2022 Sep 26;13:5641. doi: 10.1038/s41467-022-33419-1 (PMC9513080; doi:10.1038/s41467-022-33419-1)
Supplement: Supplementary file 5 — Reporting Summary [file 41467_2022_33419_MOESM5_ESM.pdf]

Corresponding author(s): Andrew Benson
Last updated by author(s): 09-13-2022

Reporting Summary

Nature Portfolio wishes to improve the reproducibility of the work that we publish. This form provides structure for consistency and transparency in reporting. For further information on Nature Portfolio policies, see our Editorial Policies and the Editorial Policy Checklist.

Statistics

For all statistical analyses, confirm that the following items are present in the figure legend, table legend, main text, or Methods section.

n/a	Confirmed	
☐	☒	The exact sample size (*n*) for each experimental group/condition, given as a discrete number and unit of measurement
☐	☒	A statement on whether measurements were taken from distinct samples or whether the same sample was measured repeatedly
☐	☒	The statistical test(s) used AND whether they are one- or two-sided<br>*Only common tests should be described solely by name; describe more complex techniques in the Methods section.*
☐	☒	A description of all covariates tested
☐	☒	A description of any assumptions or corrections, such as tests of normality and adjustment for multiple comparisons
☐	☒	A full description of the statistical parameters including central tendency (e.g. means) or other basic estimates (e.g. regression coefficient) AND variation (e.g. standard deviation) or associated estimates of uncertainty (e.g. confidence intervals)
☐	☒	For null hypothesis testing, the test statistic (e.g. *F*, *t*, *r*) with confidence intervals, effect sizes, degrees of freedom and *P* value noted<br>*Give P values as exact values whenever suitable.*
☒	☐	For Bayesian analysis, information on the choice of priors and Markov chain Monte Carlo settings
☒	☐	For hierarchical and complex designs, identification of the appropriate level for tests and full reporting of outcomes
☒	☐	Estimates of effect sizes (e.g. Cohen's *d*, Pearson's *r*), indicating how they were calculated

Our web collection on statistics for biologists contains articles on many of the points above.

Software and code

Policy information about availability of computer code

Data collection	Illumina Miseq Control Software (version 2.6.21) was used for sequencing data collection.
Data analysis	Paired-end reads from the 16S data were analyzed using QIIME 2(version 2019.1).<br>A neighbor-joining tree of full-length 16S sequences from representative species was generated using MUltiple Sequence Comparison by Log-Expectation (MUSCLE) https://www.ebi.ac.uk/Tools/msa/muscle/ (Figure 1).<br>Image analysis of panicles from sorghum plants was conducted using a set of scripts for automatic seed image analysis (https://github.com/alejandropages/SLHTP) (Figure 2 and Figure 3).<br>The genetic map was constructed using ASMap package version 1.0-4 (Figure 2).<br>QTL mapping for each phenotypic trait was conducted using R/qtl package version 1.47-9 (Figure 2, Table 1,Table S2)<br>Amplicon sequence variant analysis was performed using phyloseq version 1.32.0 and vegan version 2.5-7 packages.<br>Data was visualized using R version 4.0.4 and Rstudio version ggplot2 version 3.3.3, ggpubr version 0.4.0, and Complexheatmap packages version 2.4.3 in R, and Interactive Tree Of Life (iTOL) v5.<br>The code used in the analysis can be found at https://github.com/qinnanyang/SorgRIL.

For manuscripts utilizing custom algorithms or software that are central to the research but not yet described in published literature, software must be made available to editors and reviewers. We strongly encourage code deposition in a community repository (e.g. GitHub). See the Nature Portfolio guidelines for submitting code & software for further information.

Data

Policy information about availability of data

All manuscripts must include a data availability statement. This statement should provide the following information, where applicable:

- Accession codes, unique identifiers, or web links for publicly available datasets
- A description of any restrictions on data availability
- For clinical datasets or third party data, please ensure that the statement adheres to our policy

The DNA sequencing reads for this study are available in the NCBI SRA database as project accession PRJNA801694. All ASVs were assigned with taxonomic information using pre-fitted sklearn-based taxonomy classifier SILVA database (release 132). Genotype data of the RILs can be found at Figshare: https://doi.org/10.25387/g3.6304538. Source data are provided with this paper.

Field-specific reporting

Please select the one below that is the best fit for your research. If you are not sure, read the appropriate sections before making your selection.

☒ Life sciences ☐ Behavioural & social sciences ☐ Ecological, evolutionary & environmental sciences

For a reference copy of the document with all sections, see nature.com/documents/nr-reporting-summary-flat.pdf

Life sciences study design

All studies must disclose on these points even when the disclosure is negative.

Sample size	294 lines of RIL is sufficient and typical in plant QTL analysis. A sample size calculation was not performed to determine the sample size. The sample size was determined based upon the number of availability of RIL lines. Sample size is based on previous publications using the same RIL population: Hart et al. Theor Appl Genet (2001) 103:1232–1242; and Kong et al. G3 . 2018 Aug; 8(8): 2563–2572.
Data exclusions	Taxonomic abundances (phenotypes) were filtered based on a conservative threshold of at least 100 reads per sample
Replication	The mapping was based on single growout of RILs (one replication). Each RIL X microbiome fermentation was done in triplicate. Phenotypic data from all three replicates was used for analysis. Further details about the replication have been included in the figure legends and in methods.
Randomization	For the QTL analysis, the triplicate line X microbiome replicates were randomized across twelve 96-well plates with plates as incomplete blocks. For all the other in vitro fermentation experiments, each fecal samples were allocated with all the treatment groups. And all treatment groups for the same experiment were conducted at the same time.<br>For experiment with pools of RILs, RILs were grouped by haplotype of markers linked to Tan1 and Tan2 and were randomly selected and pooled within each haplotype group.<br>For tannin complementation experiment and tannin enrichment experiment, all treatment groups were inoculated with five human fecal microbiomes with the most significant responses between tannin negative RILs and tannin positive RILs sorghum observed in in vitro fermentations (smallest p-value from PERMANOVA analysis).
Blinding	We were blinded to the genetic background of each individual line when conducting the experiment.

Reporting for specific materials, systems and methods

We require information from authors about some types of materials, experimental systems and methods used in many studies. Here, indicate whether each material, system or method listed is relevant to your study. If you are not sure if a list item applies to your research, read the appropriate section before selecting a response.

Materials & experimental systems

n/a	Involved in the study	
☒	☐	Antibodies
☒	☐	Eukaryotic cell lines
☒	☐	Palaeontology and archaeology
☒	☐	Animals and other organisms
☐	☒	Human research participants
☒	☐	Clinical data
☒	☐	Dual use research of concern

Methods

n/a	Involved in the study	
☒	☐	ChIP-seq
☒	☐	Flow cytometry
☒	☐	MRI-based neuroimaging

Human research participants

Policy information about studies involving human research participants

Population characteristics	Fresh fecal samples were obtained from 12 adult participants (3 females and 9 males, ages 23 to 41) with no history of gastrointestinal abnormalities and no prebiotic, probiotic, or antibiotic consumption within the past six months. Stool sample were collected using a commode specimen collection kit (Fisher Scientific, NH, USA). All procedures involving human subjects were approved by the Institutional Review Board of the University of Nebraska–Lincoln before initiating the study (20160816311EP).
Recruitment	Participants responded to flyers that distributed at department of Food science and Technology, University of Nebraska-Lincoln. All participants that responded were selected for the study. There was no self-selection for choosing participants.
Ethics oversight	Institutional Review Board of the University of Nebraska–Lincoln

Note that full information on the approval of the study protocol must also be provided in the manuscript.
